# Towards Safe African Swine Fever Vaccines: The *A137R* Gene as a Tool to Reduce Virulence and a Promising Serological DIVA Marker Candidate

**DOI:** 10.3390/ani14172469

**Published:** 2024-08-25

**Authors:** Andrey Koltsov, Mikhail Sukher, Sergey Krutko, Sergey Belov, Alexey Korotin, Sofia Rudakova, Sergey Morgunov, Galina Koltsova

**Affiliations:** Federal Research Centre for Virology and Microbiology, Academician Bakoulov Street 1, 601125 Volginsky, Russia; kolcov.andrew@gmail.com (A.K.); sergejjkrutko@gmail.com (S.K.); belovsergej371@gmail.com (S.B.); alescha.korotin@yandex.ru (A.K.); sofiia.rudakova@mail.ru (S.R.);

**Keywords:** African swine fever, African swine fever virus, ASFV, recombinant virus, *A137R*, p11.5 protein, pA137R protein, DIVA

## Abstract

**Simple Summary:**

African swine fever (ASF) causes serious economic losses to the global pig industry. Although several commercial vaccines have been registered in some Asian countries, the development of ASF vaccines is still actively continuing. One of the current trends is the development of a safe DIVA vaccine against ASF, the use of which will allow differentiating between infected or vaccinated pigs. To date, commercial ASF vaccines are live vaccines based on the ASFV attenuated strains with the deletion of the viral genes responsible for virulence. However, some recombinant strains with the deletions of virulence genes may have residual virulence and be unsafe. In our work, we have shown that the infection of animals with the ASFV *A137R*-deletion mutant with a dose 10 times higher than the described safe dose leads to the death of 87.5% of the infected animals. We assume that the deletion of the *A137R* gene may be additionally introduced into the genomes of candidate vaccine strains in order to reduce their reactogenicity and as an antigenic and genetic marker for the differentiation of infected and vaccinated animals (DIVA strategy).

**Abstract:**

African swine fever (ASF) is an emerging disease caused by the African swine fever virus (ASFV), which is a great threat to the swine industry worldwide. Currently registered vaccines that have demonstrated protection against the homologous ASFV strains are live attenuated vaccines based on recombinant ASFV strains with the deletions of virulence-associated genes. In this study, we evaluated the deletion of the *A137R* gene in the ASFV virulent Stavropol_01/08 strain isolated in Russia in 2008. Our animal experiment results demonstrated that the deletion of the *A137R* gene did not lead to the full attenuation of this strain, and increasing the dose of the *A137R*-deletion mutant during infection led to the death of 87.5% of the infected animals. In this report, we also demonstrated that immunofluorescence (IFA) and Western blotting assays based on the recombinant p11.5 protein can be used to detect antibodies in animals infected with the attenuated ASFV variants of several genotypes/serotypes. Both assays were specific to ASFV p11.5 protein and showed negative results when examining the sera of the non-infected animals or those infected with the *A137R*-deletion mutant. Therefore, we propose to use the p11.5 protein along with other previously proposed ASFV proteins, such as CD2v, as negative antigenic DIVA markers for an attenuated ASF vaccine.

## 1. Introduction

African swine fever (ASF) is a viral hemorrhagic disease of domestic pigs with a mortality rate that reaches 100% [1,2,3]. African swine fever virus (ASFV), the causative agent of ASF, is a large DNA virus that belongs to the *Asfarviridae* family [4]. Genotyping based on the *B646L* gene (encoding the p72 protein) has made it possible to identify at least 24 genotypes of ASFV [5,6,7,8,9]. Additionally, a new classification of ASFV was performed considering the entire encoded proteome, which classified 220 ASFV isolates/strains into seven distinct biotypes [8].

ASFV of various genotypes is distributed in most parts of Africa, where it is maintained in a sylvatic cycle between the soft ticks of the genus *Ornithodoros* and natural mammalian hosts, primarily warthogs (*Phacochoerus* sp.) [10,11]. After the ASF genotype II virus was imported from East Africa to Georgia [12], it began to circulate in Eastern Europe (since 2007), in the European Union (since 2014), in Asia (since 2018), in America (since 2021), and Oceania (since 2020) (the World Organization for Animal Health (WOAH)) [13].

The vaccines that have currently demonstrated full or partial protection against the homologous ASFV strain are based on three different variants of the virus: naturally occurring low-virulent ASFV strains [14,15,16,17], attenuated by consecutively passing in homologous or heterologous cell lines [18,19,20,21,22], and recombinant ASFV strains with the deletion of the virulence-associated genes [23,24,25,26,27,28]. The main problem with using naturally attenuated ASFV strains is the high risk of developing chronic or persistent infection, as well as secondary bacterial or viral infections in inoculated pigs [14,15,17]. Compared with targeted deletion of the virulence-associated genes from the viral genome, serially passaging is a longer process with an unpredictable result. Thus, it was shown that ASFV strains that are serial passed in non-primary cell lines (such as Vero, SV-1, and A4C2 cells) become attenuated, but they lose their ability to confer protection [18,29]. Thus, currently, the most promising and fastest method for generating LAV vaccines against ASF is obtaining recombinant ASFV strains with the deletion of the virulence-associated genes.

One of the genes involved in the virulence of ASFV is the late-expressed gene *A137R*, encoding the viral structural p11.5 protein [30,31]. The *A137R* gene is highly expressed in ASF-infected cells [32,33]. The deletion of this gene, as well as some other genes associated with ASFV virulence, such as *E184L* [34], *EP402R* [25,27,35], and *I177L* [26] and the multigene families *MGF360* and *MGF505* [23,27,36] does not lead to the loss of protection against the challenge of the parental ASFV [31].

Based on the results of the transcriptome sequencing of the primary porcine alveolar macrophages (PAMs) infected with the wild-type ASFV HLJ/2018 strain and *A137R* gene-deleted ASFV mutant, it was demonstrated that p11.5 (pA137R) suppresses type I IFN production [37]. The mechanism of suppression is due to the p11.5 protein, which inhibits the cGAS-STING-mediated IFN-β signaling pathway. pA137R interacts with TBK1 and promotes the autophagy-mediated lysosomal degradation of TBK1. This process leads to the inhibition of IRF3 nuclear translocation [37].

Li et. al. (2024) presented results showing that the ASFV p11.5 protein is capable of self-oligomerizing to form a dodecahedron cage composed of 60 promoters [38]. The authors suggest that this unique property of the protein can be used in the development of heterologous vaccines based on molecular scaffolds from the p11.5 protein for nanobiology applications [38].

Considering its potentially high immunogenicity and the important role of the p11.5 protein in viral virion assembly, as well as the high expression level of the *A137R* gene during viral replication, some researchers have considered this gene/protein as a target for diagnostic tools. A highly sensitive SYBR Green-based real-time PCR assay for the detection of ASFV which targets the *A137R* gene was developed [39]. In addition, the p11.5 protein is an interesting potential detection target for serological analysis. Therefore, an indirect serological enzyme-linked immunosorbent assay (ELISA) based on recombinant p11.5 protein and ASFV pA137R-specific mAb was developed [40,41]. Additionally, this mAb can be used in ELISA, IHC, and immunochromatographic strip assays. 

One way to overcome the extremely high risks of using live attenuated vaccines against ASF is the development of DIVA vaccines and accompanying tests. DIVA strategies as a differentiation method between infected and vaccinated animals may in the future become significant tools for the disease control and eradication of ASF.

Given the central importance of identifying virulence genes and antigenic markers for differentiating between infected and vaccinated animals in the ASF vaccine development, we aimed to investigate the possibility of using the *A137R* gene simultaneously to reduce ASFV virulence and as a marker for the DIVA vaccine. To address these questions, we obtained a recombinant ASFV genotype II lacking the *A137R* gene (ΔA137R_Stavropol_01/08 strain) and studied its virulence by infecting pigs with 10^3^ HAD_50_. We also tested porcine sera using WB and IFA and demonstrated that pA137R protein is a highly immunogenic protein, although anti-pA137R antibodies are completely absent in animals infected with the ASFV ΔA137R_Stavropol_01/08 strain.

Thus, in this study, we showed that increasing the dose of the ASFV recombinant strain with an *A137R* gene deletion when infecting animals leads to their death, although previously the *A137R*-deletion mutant was considered a promising candidate for a live-attenuated vaccine [31]. Despite the residual virulence of the *A137R*-deletion mutant, we assume that the deletion of the *A137R* gene can be additionally introduced into the genomes of candidate vaccine strains in order to reduce their reactogenicity and serve as a negative antigenic marker for the differentiation of infected and vaccinated animals (DIVA strategy).

## 2. Materials and Methods

### 2.1. Cell Cultures and Viruses

ASF Stavropol_01/08 virulent virus (genotype II, serogroup 8) reference strains of serotypes 1, 2, 3, 4, and 8 (L57, K49 (Congo-v), M78, F32 (France-v), and Rhodesia) [42] were obtained from the collection of the FRC of Virology and Microbiology (FRCVM, Volginsky, Russia). The ASFV Stavropol_01/08 strain was originally isolated from a domestic pig in the Stavropol region of Russia in 2008. 

To standardize conditions, the viral stocks (the parental and the recombinant strains) were produced and titrated in the primary cultures of porcine macrophages obtained using the same donors. The primary cultures of porcine macrophages were prepared from blood using Ficoll solution with a density of 1077 g/cm^3^ (PanEco, Moscow, Russia). The obtained cells were cultured in 96-well (1–3 × 10^6^ cells/well) or 48-well plates (4 × 10^6^ cells/well) (Guangzhou Jet Bio-Filtration Co., Guangzhou, China) in media containing RPMI 1640 (PanEco, Moscow, Russia) with the addition of 10% (*v*/*v*) fetal bovine serum (Cytiva, Marlborough, MA, USA), 30% (*v*/*v*) plasma, and antibiotic gentamicin 0.02 mg/mL (PanEco, Russia) in a CO_2_ incubator (temperature 37 °C, 5% CO_2_) for 24 h. The adherent cells were washed with the same medium and used in experiments after 36–48 h.

COS-1 cells were kindly provided by C. Gallardo (CISA-INIA, Valdeolmos, Spain). The cells were grown in Dulbecco’s Modified Eagle Medium/Nutrient Mixture F-12 (DMEM/F-12) (PanEco, Russia), supplemented with the antibiotic gentamicin 0.01 mg/mL (PanEco, Russia) and 10% fetal bovine serum (Cytiva, USA) at 37 °C with 5% CO_2_.

The viruses (parental and recombinant) were titrated in 96-well plates based on the presence of hemadsorption in the primary cultures of porcine macrophages (in the presence of pig erythrocytes). Titers were expressed as the average infectious dose of hemadsorption doses (HAD_50_) using the Reed–Muench method [43].

The growth curves of the parental ASFV Stavropol_01/08 strain and its *A137R*-deletion mutant were measured after the infection of the primary cultures of porcine macrophages in 48-well plates as described previously [44]. The cultures were infected with viruses at an MOI of 0.1. The infected cells were collected at 0, 24, 48, 72, 96, and 120 h after infection (hpi) and stored frozen at ≤−50 °C until lysates were used for ASFV titration in the primary cultures of porcine macrophages. 

### 2.2. Construction of A137R-Deletion Mutant

The recombinant *A137R*-deletion mutant (ΔA137R_Stavropol_01/08) was constructed from the virulent ASFV Stavropol_01/08 strain by homologous recombination using a recombination cassette consisting of the reporter *EGFP* gene (under the control of the *A137R* gene promoter), flanked by two recombination arms as described earlier [20,22]. COS-1 cells were used to obtain recombinant clones by infecting with the parent virus (at MOI = 5) and subsequently transfecting with the recombination cassette 2 h after infection. The infection/transfection of the cell line was carried out in 6-well plates (Guangzhou Jet Bio-Filtration Co., China).

The selection of the *A137R*-deletion mutant was based on reporter fluorescence and was carried out in the primary cultures of porcine macrophages by the method of limited dilutions using the ZOE fluorescent cell imager (Bio-Rad Laboratories, Hercules, CA, USA). The purity of the selected virus was confirmed using the SYBR Green PCR protocol with 2 different pairs of the *A137R* gene-specific primers as described previously [31,39], and the master mix 5X qPCRmix-HS SYBR (Evrogen, Moscow, Russia). The sequence accuracy of the recombinant virus was confirmed by the sequencing of the recombination site (position 54945-56426 in the genome of ASFV genotype II Georgia 2007/1, GenBank NC_044959.2). The sequencing was performed using the Sanger method on a 3130xl Genetic Analyzer (Thermo Fisher, Waltham, MA, USA).

### 2.3. Animal Experiments and Ethics Statement

All the animal procedures were conducted in accordance with Russian legislation on the protection of animals used for scientific purposes, and the experiments were approved by the Research Ethics Committee of the Federal Research Center of Virology and Microbiology, Russia (№01/2024).

Specific pathogen-free piglets of the Large White pig breed, not vaccinated against any infections, were received from the Experimental Animal Preparation Sector of the FRCVM. To confirm the absence of swine pathogens (pseudorabies virus (PRV), classical swine fever virus (CSFV), ASFV, porcine reproductive and respiratory syndrome virus (PRRSV), and porcine circovirus types 2 and 3 (PCV2 and 3), transmissible gastroenteritis virus (TGEV), porcine epidemic diarrhea virus (PEDV)) in the animals, samples collected from all the pigs before the experiment were tested by real-time PCR using commercial kits (VectorBest, Novosibirsk, Russia).

A total of 12 white pigs (males (*n* = 8), females (*n* = 4)) aged 2.5–3 months (weighing 18–20 kg) were obtained. The piglets were randomly divided into two groups. Each group of animals was kept in isolated rooms throughout the experiment. The acclimatization period for each group was 10 days, after which the animals were infected. The pigs from group 1 (*n* = 8) were inoculated intramuscularly with recombinant ASFV ΔA137R_Stavropol_01/08. Pigs from group 2 (*n* = 4) were inoculated intramuscularly with the parental ASFV Stavropol_01/08. Both the viruses were injected at a dose of 10^3^ HAD_50_ (Supplementary Materials, Figure S1). 

The pigs’ rectal body temperature and clinical signs were measured daily throughout the experiment. A clinical evaluation of ASF was performed in 4 different categories (behavior, neurological signs, defecation, and body temperature). The clinical signs were assigned numerical values based on the severity and significance as described in [45]. The total score was recorded as a clinical score. The clinical scores were recorded daily for each pig.

The animals that reached a pre-moribund state were euthanized to minimize animal suffering. Time to death and survival were recorded as described earlier [46]. The day-to-fever value corresponded to the first-day post-challenge when rectal body temperature was recorded to rise above 40 °C. To assess viremia, blood samples were taken from the jugular vein at 0, 3, 5, 7, 14, 21, and 28 days post-inoculation (dpi) (Supplementary Materials, Figure S1). Five tissues (lung, liver, spleen, mesenteric, and submandibular lymph nodes) were taken from animals during autopsy and frozen at −70 °C.

The viral load in the blood and organ samples of the infected animals was assessed based on the presence/absence of the ASFV genome using the *B646L* gene-based qPCR assays as described above. DNA from the blood and tissue samples as well as from infected cell culture was isolated using the ExtractDNA Blood kit (Evrogen, Moscow, Russia) according to the manufacturer’s instructions.

ASFV ELISA assays (IDScreen^®^ African Swine Indirect, Grabels, France) were performed as recommended by the manufacturer using the serum collected at 0, 7, 14, 21, and 28 dpi. This commercial ELISA kit is based on three recombinant antigens: P32, P62, and P72. The results are expressed as S/P%, where S = optical density of the sample—optical density of the negative control, and P = optical density of the positive control—optical density of the negative control.

### 2.4. ASFV pA137R-Coding Sequence Amplification, Cloning and Expression

The target ASFV DNA fragment, 410 bp in length (full-length *A137R* gene of the ASFV Stavropol_01/08 strain), was inserted into the BamHI and XhoI sites of the expression vector pEF1a_3HA to generate pEF1a_A137R_3HA by a T4 DNA ligase (NEB, Ipswich, MA, USA) binding reaction after cleavage with relevant restriction enzymes (NEB, USA). The ASFV *A137R* gene-specific primers (Forward: TAA GGC CTC TGG ATC CAT GGA AGC AGT TCT TAC CAA ACT C; Reverse: TAT ATA TCT AGA GCC TTC TTT GAT ATT CAT CTT GCC) were used for the amplification of the target DNA. The HA-tag was added to the C-terminal region of protein p11.5. The expression vector pEF1a_3HA was kindly provided by Kholod N. *Escherichia coli* XL1-Blue competent cells (Evrogen, Russia) were used for transformation. The recombinant plasmids were tested by PCR and sequenced using the Sanger method to verify the nucleotide sequence.

The resulting recombinant plasmid pEF1a_A137R_3HA was used to transfect COS-1 cell cultures for the transient expression of the p11.5 protein. The commercial transfection reagent GenJect-40 (Molecta, Moscow, Russia) was used according to the manufacturer’s instructions. The confirmation of the expression of HA-tagged recombinant p11.5 proteins was carried out by immunofluorescence assay and Western blot using anti-HA antibodies (primary antibodies) (Thermo Fisher Scientific, Waltham, MA, USA) and goat anti-mouse antibodies (secondary antibody) (Thermo Fisher Scientific, USA).

### 2.5. Testing of Pig Sera by Indirect Immunofluorescence Assays and Western Blot

A total of 54 sera samples were used in this study. Of these, 30 samples were collected from the animals infected with different ASFV strains and pre-tested as positive or doubtful (S/P 30–40%) using a commercial ELISA kit (IDScreen^®^ African Swine Indirect, Grabels, France) as described previously. Four samples were reference hyperimmune sera against ASFV serotypes 2, 3, 4, and 8 which were obtained from the FRCVM collection. Twenty sera samples collected from animals uninfected with the ASFV were used as negative controls. All the negative samples were also screened by ELISA (IDScreen^®^ African Swine Indirect, Grabels, France).

For the immunofluorescence assays, COS-1 cells were cultured in 96-well plates. After 20 h, the cells were transfected with plasmid pEF1a_A137R_3HA using the commercial transfection reagent GenJect-40 (Molecta, Russia) according to the manufacturer’s instructions. The cells were incubated for 24 h and then washed with phosphate-buffered saline (PBS), fixed with a mixture of acetone and ethanol (30:70) at −20 °C for 10 min, washed with PBS twice, and rinsed with shaking for 10 min. The cells were then blocked with PBS containing 2% BSA at 37 °C for 30 min. All the serum samples were diluted 1:50, 1:100, and 1:1000 and incubated with cells at 4 °C overnight, and then washed with PBS three times. The cells were incubated with Alexa Flour 488 conjugated goat anti-pig secondary antibodies (Jackson ImmunoResearch, Bar Harbor, ME, USA) at 37 °C for 1 h, and then washed three times RBS and observed under the ZOE fluorescent cell imager (Bio-Rad Laboratories, USA).

Some samples (*n* = 28) were randomly selected and analyzed by Western blot. Briefly, the samples were separated on 12% polyacrylamide gel. The separated proteins were transferred onto a nitrocellulose membrane using the Bio-Rad Mini Trans-Blot Cell (Bio-Rad, USA) for Western blot. The membrane was washed three times in phosphate-buffer saline containing 0.05% Tween-20 (PBS-T) with shaking, then blocked with 5% non-fat milk in PBS-T at 4 °C for 2 h, and incubated with porcine sera samples diluted to 1:100 in PBS-T overnight at 4 °C. The membranes were washed three times with PBS-T and incubated with anti-pig secondary antibodies (Rockland Immunochemicals, Pottstown, PA, USA) for 1 h at 37 °C, and then washed three times with PBS-T. Protein bands were detected with the Clarity™ Western ECL Substrate (Bio-Rad, USA).

### 2.6. Hemadsorption Inhibition (HAI) Assays

Hemadsorption inhibition (HAI) assays were performed in the swine macrophages using ASFV parental strain and deletion mutant, hyperimmune reference antisera prepared to serotypes 1, 2, 3, 4, 8, and data reference strains serotypes as described previously [42,47]. Briefly, HAI assays were carried out in 96-well plates (Guangzhou Jet Bio-Filtration Co., China). Cells were infected with each virus (50 μL) at a dose of 10^3^ HAD_50_ per well and incubated at 37 °C with 5% CO_2_ overnight. The two-fold dilutions of hyperimmune sera (50 μL) were added to the infected cells and incubated for 4 h, and then a 1% suspension of porcine erythrocytes (20 μL) was added to the cells. The results of the HAI assay were recorded after 16–20 h by microscopy.

### 2.7. Statistical Analysis

The comparison of survival curves between the groups of pigs was determined by the log-rank (Mantel–Cox) test and the Gehan–Breslow–Wilcoxon test. The growth kinetics data are presented as the mean ± standard deviation (SD) from three independent experiments. The error bars represent standard deviations at each point. The statistical analysis of viral loads in the blood and in the organs was performed using the one-way analysis of variance (ANOVA). All the statistical analyses were performed using the GraphPad Prism software version 8.0.1 (GraphPad Software, San Diego, USA). Comparisons showing a *p*-value of <0.05 were considered statistically significant. In the figures, the asterisks denote significant differences (* *p* < 0.05; ** *p* < 0.01; *** *p* <0.001; **** *p* < 0.0001), while “ns” indicates non-significance.

## 3. Results

### 3.1. Construction of A137R-deletion Mutant

**Figure 1:**
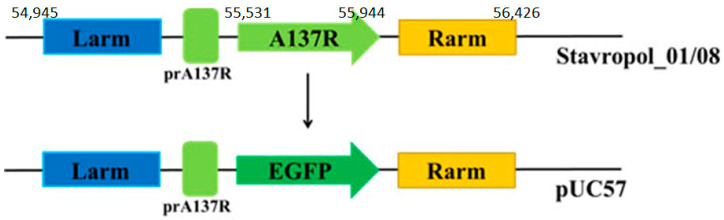
A schematic diagram representing the target site for the deletion of the *A137R* gene in the genome of the virulent strain of the ASFV Stavropol_01/08 (genotype II, serogroup 8). The position of the *A137R* open reading frame, left arm (Larm), and right arm (Rarm) are indicated based on the positions of these fragments in the ASFV strain Georgia 2007/1 genome (GenBank NC_044959.2).

In order to confirm the important role of the p11.5 protein in ASFV virulence, a recombinant strain with a deletion of the *A137R* gene was created based on the virulent strain of the ASFV Stavropol_01/08 (genotype II, seroimmunotype 8). The *A137R* deletion mutant (ΔA137R_Stavropol_01/08) was constructed by homologous recombination using a recombination cassette. The recombination cassette included the *EGFP* reporter gene under its own promoter of the *A137R* gene, flanked by two recombination arms (*A179L* and *F317L* genes), and was based on the plasmid vector pUC57 (Figure 1). The COS-1 cells were used to obtain recombinant clones by infection with the parent strain of the ASFV Stavropol_01/08 (at MOI = 5) and subsequent transfection with the recombination cassette. The selection of the *A137R* deletion mutant was carried out in primary cultures of porcine macrophages using the limited dilution method. The recombinant clones were screened for the presence of the EGFP reporter fluorescent protein (Supplementary Materials, Figure S2C). 

To detect the presence of the “wild type” (virulent strain Stavropol_01/08), the recombinant clones were tested using two different pairs of primers complementary to the *A137R* gene sequence [31,39] using the SYBR Green PCR, starting from the fifth round of limiting dilutions. The pure ASFV ΔA137R_Stavropol_01/08 strain was obtained after nine rounds of limiting dilutions. The sequencing confirmed the replacement of the *A137R* gene with the *EGFP* gene and the absence of additional mutations in the recombination site (position 54945–56426 in the genome of the ASFV II genotype Georgia 2007/1, GenBank NC_044959.2). EGFP expression was confirmed by Western blot using an anti-GFP polyclonal antibody (Santa Cruz Biotechnology, Texas, USA) when testing the samples of primary swine macrophages infected with ASFV recombinant ΔA137R_Stavropol/01/08 strain (Supplementary Materials, Figure S2D).

As expected, the same HAI serological specificity was observed for the parental virus Stavropol01/08 and its *A137R*-gene deletion mutant upon interaction with serogroup 8 reference antisera. Reference sera against other serotypes did not inhibit hemadsorption of the Stavropol01/08 and ΔA137R_Stavropol_01/08) strains (Supplementary Materials, Table S1).

### 3.2. In Vitro Replication and Growth Kinetics of A137R-Deletion Mutant in Primary Cultures of Porcine Macrophages

The recombinant ASFV strain ΔA137R_Stavropol_01/08 exhibited normal replication and hemadsorption characteristics in the primary cultures of the porcine macrophage cells. The hemadsorption pattern of the strain was identical to that of the parental strain Stavropol_01/08 (Supplementary Materials, Figure S2A,B). The recombinant strain ΔA137R_Stavropol_01/08 demonstrated the ability to replicate efficiently in the primary swine macrophage cell culture, accumulating in titers of 6–7 log10 HAD_50_/mL.

**Figure 2:**
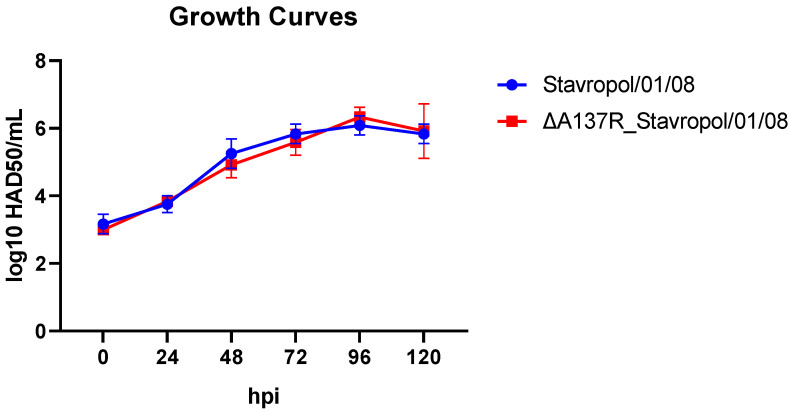
The growth kinetics of the ASFV parental Stavropol_01/08 virus (blue line) and the ASFV recombinant ΔA137R_Stavropol_01/08 strain (red line) in vitro in a primary cell culture of porcine macrophages. The multistep growth curves are given as log10 HAD_50_/mL. The growth kinetics data are presented as the mean ± standard deviation (SD) from three independent experiments. The error bars represent standard deviations at each point. The analysis was performed using the GraphPad Prism software version 8.0.1.

To study the role of the p11.5 protein in the ASFV replication in the primary cultures of porcine macrophages, the in vitro multistage growth curves of the viruses were constructed. The cells were infected with ASFV parental Stavropol_01/08 or ASFV recombinant ΔA137R_Stavropol_01/08 at a multiplicity of infection (MOI) of 0.1. At 0, 24, 48, 48, 72, 96, and 120 h post-infection (hpi); the ASFV replication was quantified by titration in the primary cultures of porcine macrophages and expressed as log10 HAD_50_/mL. The multiplicity of infection was set at 0.1 to allow for multiple cycles of virus replication during the experiment. To confirm the results obtained, three independent experiments were conducted. The mean values were used for analysis. No significant differences in replication kinetics were observed until the end of the experiment (120 hpi) (Figure 2).

These results indicated that the deletion of the *A137R* gene did not significantly affect the virus replication in the primary cell cultures.

### 3.3. Replication In Vivo and Virulence in Susceptible Animals of A137R-Deletion Mutant

In order to further study the biological properties (virulence) of the recombinant strain ΔA137R_Stavropol_01/08 and to perform a comparative analysis with the original virulent strain Stavropol_01/08, an in vivo experiment was conducted. 

Previously, Gladue et.al. (2021) [31] showed that the inoculation of animals with 10^2^ HAD_50_ doses of ASFV-G-ΔA137R did not lead to the death of the infected animals, so the purpose of this work was to determine the virulence of a similar strain with a deletion of the *A137R* gene when infecting animals with a higher dose (10^3^ HAD_50_). Notably, in Gladue’s study, the deletion mutant was obtained from the ASFV Georgia 2010 (ASFV-G) strain assigned to genotype 2 and serogroup 8, and probably genetically similar to the Stavropol_01/08 strain used in this study.

A total of 12 pigs (males/females) aged 2.5–3 months (weighing 18–20 kg) were used. To confirm the absence of the swine pathogens (PRV, CSFV, ASFV, PRRSV, PCV2, PCV3, TGEV, and PEDV), the samples collected before the experiment were tested by real-time PCR. None of the samples tested contained fragments of the porcine viral genomes.

The animals from group G1 (*n* = 8) were inoculated intramuscularly with the recombinant ASFV strain ΔA137R_Stavropol_01/08 at a dose of 10^3^ HAD_50_. The animals from the second group G2 (*n* = 4) were inoculated intramuscularly with the ASFV parental Stavropol_01/08 at a similar dose (10^3^ HAD_50_). The reverse titration of the material used to infect the animals demonstrated that all the pigs were inoculated with material containing 10^3^ HAD_50_, which was consistent with the expected titer. 

**Figure 3:**
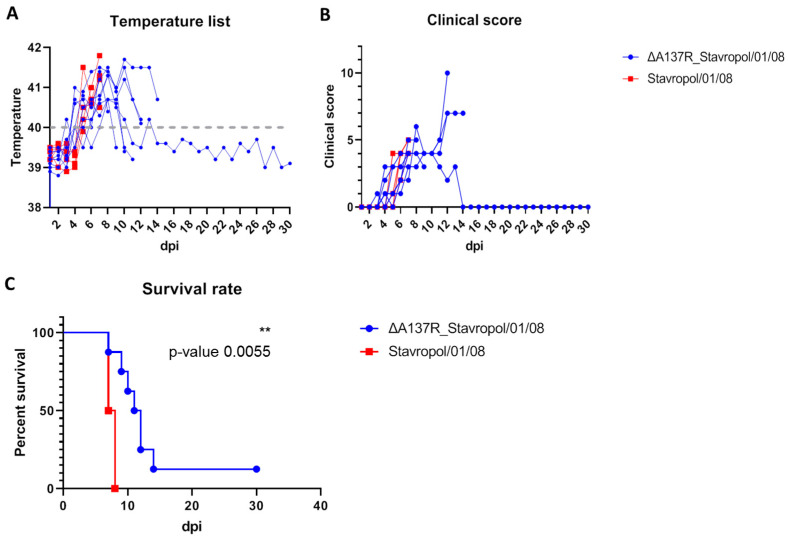
Body temperature (**A**), clinical signs (**B**), and lethality kinetics (**C**) in the pigs inoculated with ASFV parental Stavropol_01/08 strain (red line) and ASFV recombinant ΔA137R_Stavropol_01/08 strain (blue line for the animals that did not recover and green line for the surviving animal). The dashed line represents the fever cutoff (40 °C) (**A**). Data on body temperature and data on clinical assessment are presented as individual values for each animal. The comparison of survival curves between groups of pigs was determined by both the log-rank (Mantel–Cox) test and the Gehan–Breslow–Wilcoxon test (** *p* < 0.01). The analysis was performed using the Graphpad Prism software (version 8.0.1).

The mortality rate of the animals in group 2 was 100%, as anticipated. All the animals infected with the Stavropol_01/08 strain died or were found in a dying state and euthanized within 7–8 dpi with clinical signs characteristic of the acute form of ASF (Figure 3C, Table 1). Fever was observed in these animals four days after infection (Figure 3A). Hyperthermia was accompanied by a refusal to eat, apathy, disorientation, and cyanosis on the tips of the ears and tail (Figure 3B).

From day 2 to day 5 after inoculation with the ASFV recombinant ΔA137R_Stavropol_01/08 strain, the animals exhibited a fever (Figure 3A, Table 1, Supplementary Materials, Table S2). In addition, a reduction in activity and appetite was observed in conjunction with the fever (Figure 3B). At subsequent time points, the animals exhibited signs of disorientation in space and slight cyanosis at the tips of the ears and tail. The majority of infected animals exhibited signs of illness between days 4 and 11 post-infection (Figure 3C, Table 1, Supplementary Materials, Table S2). The animals exhibited clinical signs indicative of an acute and subacute course of the disease. Note that the animals from group 1 exhibited a longer life expectancy (10.7 dpi) compared to the pigs from group 2 infected with the ASFV parental Stavropol_01/08 strain (7.5 dpi) (Table 1). One animal from the G1 group survived the infection and was euthanized at the conclusion of the observation period (30 dpi). This animal exhibited a fever from 3 to 9 dpi. Slight hyperthermia was also observed in this animal at 12 dpi. There were no clinical signs of illness in this pig after 12 days. Consequently, the mortality rate in this group was 87.5% (Table 1, Supplementary Materials, Table S2). The difference in the survival of the infected animals was statistically supported (*p* < 0.01) using two independent statistical analyses.

**Figure 4:**
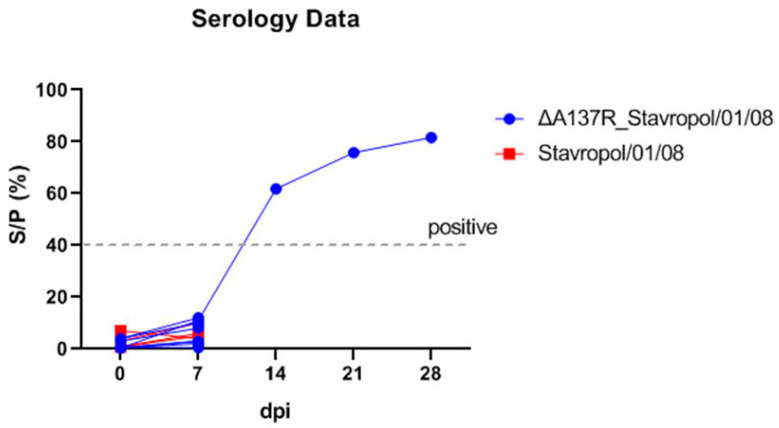
The study of humoral immune response in the pigs inoculated with the ASFV recombinant ΔA137R_Stavropol_01/08 strain (blue line) and the ASFV parental Stavropol_01/08 strain (red line) based on ELISA results. The dashed line represents the cut-off value, above which the values were considered positive (in accordance with the manufacturer’s instructions). The results of the humoral immune response assay in the pigs are expressed as S/P% and presented as individual values for each animal. The data points beyond 7 dpi are presented for the animal that survived until the end of the study. The analysis was conducted using the GraphPad Prism software (version 8.0.1).

The blood sera collected at 0, 7, 14, 21, and 28 dpi were tested for the presence of antibodies against the ASFV by ELISA using the ID Screen kit (ID Vet, Grabels, France). The ELISA results demonstrated that all the sera sampled at 0 and 7 dpi were negative. The sera of the surviving pig from group G1 infected with the ASFV recombinant ΔA137R_Stavropol_01/08 strain were found to be positive from 14 to 28 dpi (Figure 4). 

To evaluate the role of the *A137R* gene deletion in virus replication in vivo in the host, the blood and organ samples were analyzed by qPCR for the indirect assessment of viral load. The blood samples were collected at 3, 5, 7, 14, 21, and 28 dpi, and the tissue samples (lungs, liver, spleen, mesenteric, and submandibular lymph nodes) collected during autopsy. These samples were used for the DNA isolation and qPCR analysis.

**Figure 5:**
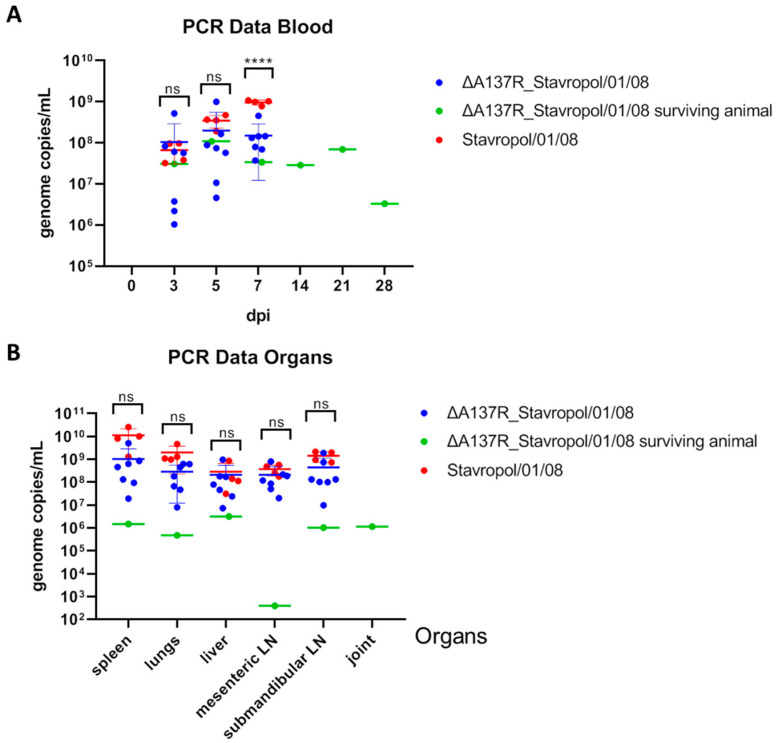
Daily viral loads in the blood (**A**) and post-mortem viral loads in the organs (**B**) in the pigs inoculated with the ASFV recombinant ΔA137R_Stavropol_01/08 strain (blue line for the animals that did not recover and green line for the surviving animal) and the ASFV parental Stavropol_01/08 strain (red line). The data on the viral load are presented in the number of viral genome copies in 1 mL in the form of individual values for each animal. The blue, green, and red bars indicate the mean value for each group. LN: lymph node. The analysis was performed using the Graphpad Prism software (version 8.0.1). The p-values were determined using one-way ANOVA (ns—*p* > 0.05; **** *p* < 0.0001). Data on the viral loads in the blood samples collected from 14 to 28 dpi, as well as the viral loads in the joints were not analyzed using one-way ANOVA due to a lack of sufficient samples.

The maximum viral genome copy values were observed at 5 and 7 dpi (Figure 5A, Table 1), although high values were also detected at 3 dpi. No statistically significant differences were observed in the viral load values in the blood (at 3–5 dpi) of the animals infected with the ASFV parental Stavropol_01/08 strain and the animals infected with the ASFV recombinant ΔA137R_Stavropol_01/08 strain (Figure 5A). Some differences (about a 10-fold difference) in the viral load were observed in the blood of the infected animals at 7 dpi (Figure 5A). It was statistically supported (*p* < 0.0001) when viral load values in the blood between the groups of pigs infected with parental or recombinant viruses were compared using the one-way ANOVA statistical analysis.

Interestingly, high values (3.31 × 10^6^) of viral genome copies were also observed in the blood of the surviving animal after infection with the recombinant strain, even at a late stage post-infection (28 dpi) (Figure 5A, Supplementary Materials, Table S2), indicating the presence of high titers of the virus in the blood despite the absence of clinical signs of the disease.

In both G1 and G2 groups, the fallen animals had characteristic pathological changes in the organs, consistent with acute ASF, including an enlarged spleen with a change in its color, serous hemorrhagic pneumonia, hemorrhagic lymphadenitis, as well as hemorrhages in the lungs, kidneys, liver, and intestines and so on. In the surviving pig infected with the ASFV recombinant ΔA137R_Stavropol_01/08 strain, the enlargement of the spleen (splenomegaly) with the formation of infarcts (dark areas) on the edges of the organ was observed. Additionally, non-significant dot hemorrhages were observed in the lungs. Hemorrhagic lymphadenitis was observed in the mesenteric lymph node area. No alterations were observed in the submandibular lymph nodes, liver, joints, gastric mucous membranes, or intestinal sections. Consequently, although the animal was clinically healthy, the replication of the virus in its body led to the development of pathological changes in tissues, which may indicate the development of a chronic form of the disease.

There were no statistically significant differences in the viral load in the organs between the groups. High levels of viral load were demonstrated in all the organs of the animals that did not recover (Figure 5B, Table 1). Thus, for the G1 group, the values ranged from 7.35 × 10^6^ to 5.12 × 10^9^ genome copies/mL, and for the G2 group, the values ranged from 3.14 × 10^7^ to 2.56 × 10^10^ genome copies/mL (Supplementary Materials, Table S2). The qPCR data are in agreement with the data from the pathological examination, which indicated numerous changes in the organs of the infected animals. The lowest values of viral load in organs were characteristic of the surviving animal (3.97 × 10^2^–3.17 × 10^6^ genome copies) (Supplementary Materials, Table S2). One of the clinical signs of the chronic form of ASF may be joint involvement. Despite the absence of changes in the joints, the ASFV genome was detected in the joint fluid of this animal (Figure 5B).

### 3.4. ASFV p11.5 Protein Expression and Sera Sample Testing by Recombinant p11.5 Based IFA and Western Blot

The expected DNA fragment of approximately 430 bp, corresponding to the full-length *A137R* gene without a stop codon of the ASFV Stavropol_01/08 strain, was amplified by PCR using primers containing BamHI and XhoI restriction sites, respectively. The expression vector pEF1a_3HA containing the eukaryotic translation elongation factor 1 alpha (EF-1α, gene symbol EEF1A1) promoter sequence and the HA-tag sequence was used to generate the pEF1a_A137R_3HA plasmid (Figure S3). Since the N-terminal domain of *A137R* is crucial and sufficient for mediating the assembly of the dodecahedron [38], the HA-tag was fused to the C-terminal region of the p11.5 protein. The constructed plasmid was transfected into the COS-1 cells for the p11.5 protein transient expression.

**Figure 6:**
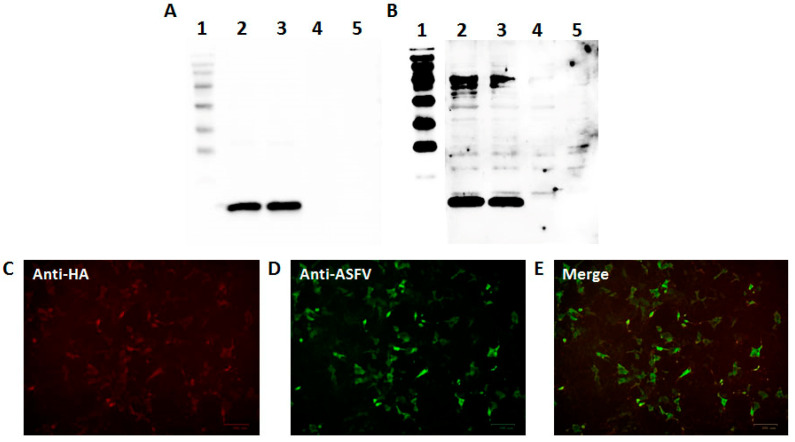
Antibody reactivity to the recombinant ASFV p11.5 protein expressed in the COS-1 cells in the Western blotting analysis and the immunofluorescence assay (IFA). (**A**) Western blotting analysis with anti-HA-tag monoclonal antibody: lane 1—MW marker; lane 2—the recombinant p11.5 protein (clone 1); lane 3—the recombinant p11.5 protein (clone 2); lanes 4–5—the COS-1 cell lysate; (**B**) Western blotting analysis with anti-ASFV hyperimmune sera in 1:100 dilution: lanes 1—MW marker; lane 2—the recombinant p11.5 protein (clone 1); lane 3—the ASFV recombinant p11.5 protein (clone 2); lanes 4–5—the COS-1 cell lysate; (**C**) IFA with anti-HA-tag monoclonal antibody. (**D**) IFA with anti-ASFV hyperimmune sera in 1:50 dilution. (**E**) Merged image. The protein pA137R distribution was visualized using appropriate secondary antibodies conjugated to Alexa Fluor 555 (red) (**C**) and Alexa Fluor 488 (green) (**D**). Both images were digitally merged (**E**) to demonstrate that the recombinant protein interacting with the anti-HA-tag monoclonal antibody and the anti-ASFV hyperimmune sera localizes to the same cells.

The recombinant p11.5 protein was expressed in the COS-1 cells as a soluble protein. The expression of the HA-tagged recombinant p11.5 protein was confirmed by the immunofluorescence assay and Western blotting with anti-HA antibodies as the primary antibodies and goat anti-mouse antibodies as the secondary antibodies (Alexa Fluor588-conjugated and HRP-conjugated, respectively). The Western blotting results demonstrated the presence of a specific band with a molecular mass of approximately 14 kDa (Figure 6A). The high efficiency of the recombinant p11.5 expression was also demonstrated by IFA (Figure 6C,E). To confirm the specificity of the HA-tagged recombinant p11.5 protein, the Western blot assay and IFA with reference hyperimmune sera against ASFV serotypes 2 were successfully performed (Figure 6B,D,E). Our results indicated that the addition of the HA-tag did not lead to significant changes in the p11.5 protein conformation.

The IFA and Western blot assays based on the recombinant p11.5 protein were used to assess whether the capabilities of reactivity to the p11.5 protein could become the basis for the development of a DIVA test to differentiate the antibody response of the animals inoculated with the *A137R*-deletion mutants from the pigs infected with ASFV encoding the p11.5 protein. A total of 54 porcine sera were tested by the immunofluorescence assays (Table 2). Of these, 34 samples were collected from the animals infected with different ASFV strains and pre-tested as positive (*n* = 28) or doubtful (inconclusive serum, S/P 30–40%) (*n* = 6) using a commercial ELISA kit. In addition, 20 ELISA-negative sera from uninfected animals were used for the analysis (Table 2). 

The result was assessed qualitatively (positive or negative), and three different dilutions of the serum (1:50, 1:100, and 1:1000) were used. All the sera of the pigs infected with ASFV containing the *A137R* gene were positive when tested by the IFA assay based on the recombinant p11.5 protein (Table 2). Although all the dilutions of these sera were positive, clearer results were obtained using a 1:1000 dilution. Conversely, no detectable antibody was found at any time point in the animals inoculated with the *A137R*-deletion mutants and the uninfected animals (Table 2). Importantly, those sera collected from the infected animals that were doubtful in ELISA were also strongly positive in IFA, which may indicate a higher sensitivity of the proposed IFA assay (Table 2).

The results of the Western blotting analysis for some positive, doubtful, and negative sera were in full agreement with the findings of IFA and showed distinct reactivity to the ASFV p11.5 protein (Table 2). Thus, both IFA and Western blotting based on the recombinant p11.5 protein make it possible to effectively and specifically detect anti-pA137R antibodies in the ASF-infected pig sera.

## 4. Discussion

Several gene-deleted recombinant ASFVs have been generated and studied recently. Some of them have only a single deletion leading to attenuation; others have several deletions simultaneously, including those located in different parts of the viral genome. The targeted virulence genes include the genes necessary for viral genome replication, genes encoding putative cellular attachment factors and proteins responsible for hemadsorption, and genes for proteins involved in modulating the host immune response. Some viral genes associated with ASFV virulence, the removal of which leads to partial attenuation, such as *E184L* [34], *EP402R* [25,35,48], *DP96R* (*UK* gene) [24], and *9GL* (*B119L*) gene [24], or full attenuation, such as *I177L* gene [26], *I1226R* gene [49], *A137R* gene [31], and genes from the multigene families *MGF360* and *MGF505* [23,27,36], are considered by researchers as the main target in genetically engineered attenuated strains obtained for ASF live vaccines. The deletions of these genes do not lead to a loss of protection against the challenge of the parental ASFV. Importantly, the safety of some deletion variants, such as ASFV strains with the deletions of the genes from the multigene families *MGF360* and *MGF505*, have been studied using both high and low doses of the virus [23,27,36]. However, other strains with the deletion of genes such as *A137R* and *E184L* have only been tested at low doses (10^2^ HAD_50_), and the safety of higher doses of these viruses is not known [31,34]. 

In this study, we constructed an *A137R*-deletion mutant (ΔA137R_Stavropol_01/08) from the virulent ASFV Stavropol_01/08 strain by homologous recombination. The complete *A137R* gene in the genome of the recombinant strain was replaced by the reporter *EGFP* gene. Our results indicated that the deletion of the *A137R* gene did not significantly affect virus replication in the primary cell culture of porcine macrophages. Our findings are consistent with those of M. Sun (2022) [37], although a study by D.P. Gladue (2021) indicated a slight decrease in the replication (10-fold) of the ASFV recombinant virus with the deletion of the *A137R* gene fragment [31]. We assume that such discrepancies are not significant and all the study data confirmed that *A137R* gene deletion is not dramatic for ASFV replication in vitro and in vivo.

In this study, we also showed that increasing the dose of the ASFV recombinant with the *A137R* gene deletion when infecting animals leads to their death. Our results demonstrated that the deletion of the *A137R* gene from the genome of the virulent Stavropol_01/08 strain did not lead to the full attenuation of this strain. The resulting recombinant strain was found to cause the death of 87.5% of the animals after infection with 10^3^ HAD_50_ and was therefore unsuitable as a prototype vaccine against ASF. The high values of the genome virus in the blood and organs of the surviving animal, the presence of pathological changes in the organs, and a long period of fever and viremia may indicate a chronic form of the disease. 

Previously, the *A137R*-deletion mutant was considered a safe vaccine strain that protected animals from challenges with the virulent ASFV Georgia strain. We assume that the differences in our results are precisely associated with an increase in the infective dose. This is indicated by the fact that despite the high mortality rate of the infected animals, we observed a longer period of life for the animals infected with the recombinant strain compared to the parental virus. 

Interestingly, Gladue et al. also indicated long-term viremia with fairly high titers of the virus in the blood (10^5^ to 10^6^ HAD_50_/mL) in most animals infected with the deletion mutant. At the same time, the virus titers decreased slightly after 11 dpi [31]. We observed similar results in the dynamics and titers of the recombinant virus in the blood of one surviving animal. Thus, although the *A137R* gene deletion reduced the ASFV virulence, it did not have a significant effect on its replication in the host.

The results of our studies indicate that the individual *A137R* gene deletion cannot be used in the development of an ASFV attenuated vaccine strain genotype II (serogroup 8); however, the deletion of this gene may be used in low-virulence field isolates or in combination with other virulence gene deletions to create safe live vaccines against ASF. The role of p11.5 protein in the ASFV virulence was shown only for genotype II viruses and should be confirmed for the other genetic and antigenic variants of the ASFV. In the future, we plan to obtain several recombinant viruses with the *A137R* gene deletion based on low virulent ASFV strains to test our hypothesis.

Our results and the data of Watanabe, M. et. al. (2023) [40] demonstrated the high immunogenicity of the p11.5 protein. We also proposed to use this protein as a negative antigenic marker to develop a DIVA-compatible vaccine candidate. This can simultaneously reduce the likelihood of complications associated with vaccination and differentiate between vaccinated and infected animals. A vigorous ASFV-specific antibody response was noted in the ASFV ΔA137R_Stavropol_01/08-inoculated animals, while all these sera were negative when tested by both the IFA and Western blot assays based on the recombinant p11.5 protein. Moreover, all the sera from the ASF-infected animals that were positive or suspicious (S/P 30–40%) in ELISA were positive when tested by these methods. Because the amino acid sequences of the *A137R* gene have high homology among the various ASFV genetic variants, the ASFV p11.5 protein is a good target in the serological investigations of ASFV. The use of this protein will enable the development and application of a single commercial kit during ASFV outbreaks regardless of the ASFV genotype or serotype involved. The main limitation of this study is the small number of serum samples available. In the future, studies with an increased number of the sera from both the animals infected with the *A137R*-deletion mutant and viruses with the *A137R* gene and the animals surviving natural ASF infection will have to be performed to determine the feasibility of using the proposed ELISA and Western blot assays for the serological ASF diagnosis. 

The p11.5 protein is not the first ASFV protein proposed as an antigenic DIVA marker for an attenuated ASF vaccine. The proteins E184L and CD2v were previously proposed as potential target markers [34,48]. However, some wild-type ASFV strains have a partially or completely deleted EP402R/CD2v gene, which may limit its use as a DIVA marker. There is currently no data on wild-type ASFV strains with a deletion of the *A137R* gene. So, we propose that the addition of the *A137R* deletion could also be included in ASF vaccine candidates to produce a potential DIVA ASF vaccine.

## 5. Conclusions

In this study, we demonstrated that the deletion of the *A137R* gene in the ASFV Stavropol_01/08 strain genome did not lead to the full attenuation of this strain. We also noted that the IFA and Western blot assays based on the recombinant p11.5 protein make it possible to detect anti-ASFV antibodies in ASF-infected pig sera. Thus, we propose that the deletion of the *A137R* gene may be additionally introduced into the genomes of candidate vaccine strains in combination with other virulence gene deletions to create safe live DIVA vaccines against ASF in the future.

## Figures and Tables

**Table 1:** Summary of the results of a comparative analysis of the virulence and viral load in pigs infected with 10^3^ of the ASFV recombinant ΔA137R_Stavropol_01/08 strain and the ASFV parental Stavropol_01/08 strain.

Group	No of Animals	Mortality		Fever		Result of ELISA	Viral Load in Blood (Max)	Viral Load in Organs (Max)
		%	TTD	%	TTF	%	Genome Copies/mL	Genome Copies/mL
ΔA137R_Stavropol_01/08	8	87.5	10.7	100	3.875	12.5	1.64 × 10^8^	5.12 × 10^9^
Stavropol_01/08	4	100	7.5	100	4	0	1.07 × 10^9^	2.56 × 10^10^

TTD, Mean time-to-death in days post-challenge, with SE in parentheses. TTF, Mean time-to-fever in days post-challenge, with SE in parentheses.

**Table 2:** Testing of porcine serum samples using ELISA, Western blotting, and IFA.

Samples	Positive Results *		
	IFA	WB	ELISA
The reference anti-ASFV hyperimmune sera	4/4	4/4	4/4
Positive porcine serum samples collected at 14–28 dpi with the ASFV attenuated strains encoding p11.5	18/18	10/10	18/18
Inconclusive porcine serum samples collected at 14–28 dpi with the ASFV attenuated strains encoding p11.5	6/6	4/4	0/6
Porcine serum samples collected at 14–28 dpi with the ASFV *A137R*-deleted mutants	0/6	0/6	6/6
Porcine negative sera	0/20	0/4	0/20

* number of positive serum samples/number of tested serum samples; IFA—immunofluorescence assay; WB—the Western blot analysis.

## Data Availability

Data are contained within the article and Supplementary Materials.

## Supplementary Materials

The following supporting information can be downloaded at: https://www.mdpi.com/article/10.3390/ani14172469/s1, Figure S1: Scheme of the experimental procedures of the inoculation and sample collection; Figure S2: Hemadsorption assays (HAs) in the primary porcine macrophages infected with the ASFV parental Stavropol/01/08 strain (A) or the recombinant ASFV ΔA137R_Stavropol/01/08 strain (B) (72 h post-inoculation); Figure S3: Analysis of the construct pEF1a_A137R_3HA for the expression of the p11.5 protein by electrophoresis and PCR; Table S1: Results of the serotyping of the recombinant ASFV ΔA137R_Stavropol_01/08 strain using reference viruses and sera (serotypes 1 (SG1), serotype 2 (SG2), serotype 3 (SG3), serotype 4 (SG4), and serotype 8 (SG8)) by hemadsorption inhibition assays; Table S2: Summary of the results of a comparative analysis of the virulence and viral loads in the animals.
