# Supplementary material for: Towards Safe African Swine Fever Vaccines: The A137R Gene as a Tool to Reduce Virulence and a Promising Serological DIVA Marker Candidate
Source: Animals (Basel). 2024 Aug 25;14(17):2469. doi: 10.3390/ani14172469 (PMC11394529; doi:10.3390/ani14172469)
Supplement: Supplementary file 1 [file animals-14-02469-s001.zip › animals-3054306-supplementary.pdf]

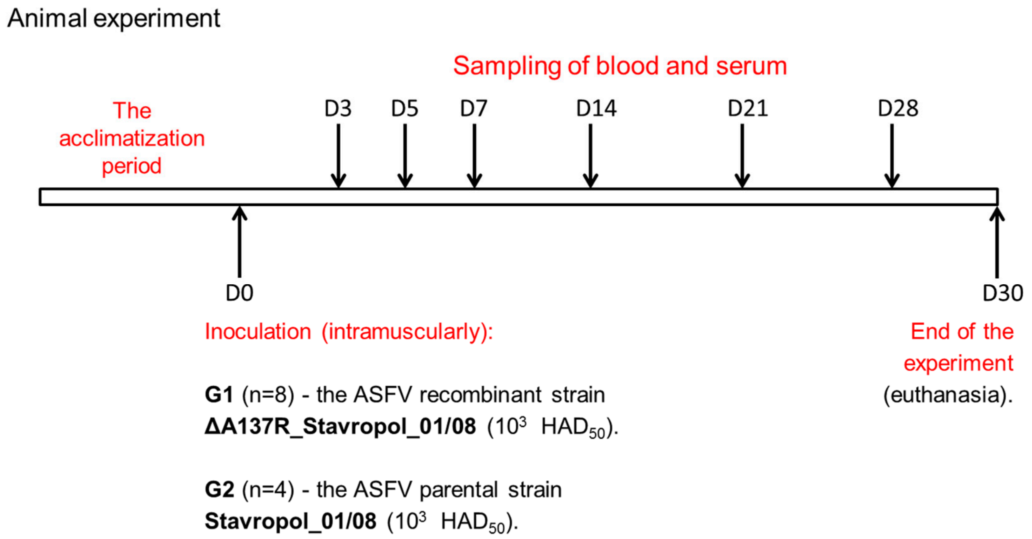

Figure S1. Scheme of experimental procedures for the inoculation and sample collection. The inoculation was performed at 0 dpi, and blood samples and serums were collected at 0, 3, 5, 7, 14, 21, and 28 dpi.

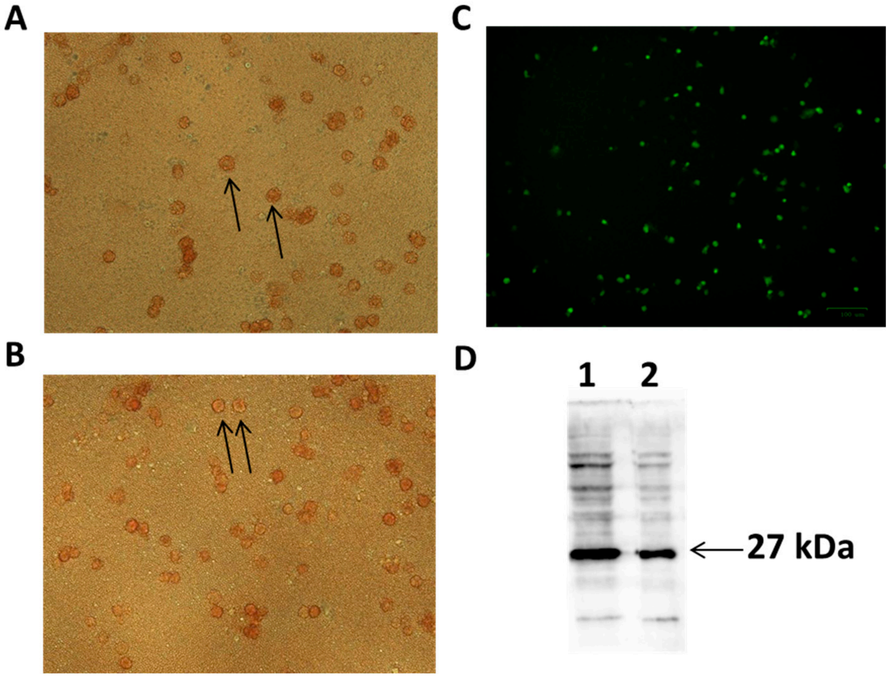

Figure S2. Hemadsorption assays (HA) in primary porcine macrophages infected with the ASFV parental Stavropol/01/08 strain (A) or the recombinant ASFV ΔA137R_Stavropol/01/08 strain (B) (72 hours post inoculation). The arrows point to the erythrocyte rosettes around ASFV-infected cells (A, B). (C) Fluorescence microscopy of the primary swine macrophages infected with the recombinant ASFV ΔA137R_Stavropol/01/08 strain (MOI = 0.1) (48 hours post-inoculation) is shown (C). (D) Western blot analysis with anti-GFP polyclonal antibody: lane 1 shows the primary swine macrophages infected with the recombinant ASFV ΔA137R_Stavropol/01/08 strain (MOI = 1) (24 hours post-inoculation); lane 2 shows the primary swine macrophages infected with the recombinant ASFV ΔA137R_Stavropol/01/08 strain (MOI = 1) (48 hours post-inoculation).

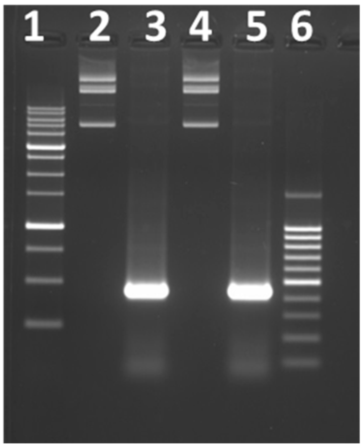

Figure S3. Analysis of the construct pEF1a_A137R_3HA for the expression of p11.5 protein by electrophoresis and PCR. Plasmid pEF1a_A137R_3HA and the specific PCR fragments were analyzed by electrophoresis in 1.5% agarose gel: lane 1 contains a 1 kb DNA ladder (Evrogen, Russia); lane 2 shows electrophoresis of plasmid pEF1a_A137R_3HA clone 1; lane 3 shows the PCR fragment of the complete A137R gene from plasmid pEF1a_A137R_3HA clone 1 tested by PCR with specific primers; lane 4 shows electrophoresis of plasmid pEF1a_A137R_3HA clone 2; lane 5 shows the PCR fragment of the complete A137R gene from plasmid pEF1a_A137R_3HA clone 2 tested by PCR with specific primers; lane 6 contains a 100 bp DNA ladder (Evrogen, Russia).

Table S1. Results of serotyping of the recombinant ASFV ΔA137R_Stavropol_01/08 strain using reference viruses and sera (serotype 1 (SG1), serotype 2 (SG2), serotype 3 (SG3), serotype 4 (SG4), serotype 8 (SG8)) by hemadsorption inhibition assays.

**ASFV strain**	**HAI titer***					
	**Anti-ASFV reference serum**					**Negative serum**
	**SG1**	**SG2**	**SG3**	**SG4**	**SG8**	
L57 (SG1)	+ 1:16	-	-	-	-	-
K49 (SG2)	-	+ 1:32	-	-	-	-
M78 (SG3)	-		+ 1:32	-	-	-
F32 (SG4)	-			+ 1:16		-
Rhodesia (SG8)	-				+ 1:32	-
Stavropol_01/08 (SG8)	-				+ 1:16-1:32	-
ΔA137R_Stavropol_01/08	-	-	-	-	+ 1:16-1:32	-

SG – serotype (serogroup) based on HAI assay results.

*Dilution of the serogroup-specific reference serum resulting in complete HAI. The results are representative of two or more independent experiments.

Table S2. Summary of the results of a comparative analysis of the virulence and viral loads in the animals that were infected with the recombinant ASFV ΔA137R_Stavropol_01/08 strain, but did not recovered; the surviving animal, infected with the recombinant ASFV ΔA137R_Stavropol_01/08 strain, and the pigs inoculated with the parental ASFV Stavropol_01/08 strain.

Group	No of Animals	Mortality		Fever		Result of ELISA	Viral Load in Blood (Min-Max)	Viral Load in Organs (Min-Max)
		%	TTD	%	TTF	%	genome copies/mL	genome copies/mL
**ΔA137R_Stavropol_01/08** the animals that did not recover	7	100	10.7	100	4.14	0	1.03×10^6 - 1.64×10^8	7.35×10^6– 5.12×10^9
**ΔA137R_Stavropol_01/08** the surviving animal	1	0	0	100	3	100	3.31×10^6 – 1.09×10^8	3.97×10^2 - 3.17×10^6
**Stavropol_01/08**	4	100	7.5	100	4	0	3.22×10^7 - 1.07×10^9	3.14×10^7 - 2.56×10^{10}

TTD, Mean time-to-death in days post-challenge, with SE in parentheses.
TTF, Mean time-to-fever in days post-challenge, with SE in parentheses.
